# Speech recognition in noise task among children and young-adults: a pupillometry study

**DOI:** 10.3389/fpsyg.2023.1188485

**Published:** 2023-06-23

**Authors:** Avital Trau-Margalit, Leah Fostick, Tami Harel-Arbeli, Rachel Nissanholtz-Gannot, Riki Taitelbaum-Swead

**Affiliations:** ^1^Department of Communication Disorders, Speech Perception and Listening Effort Lab in the Name of Prof. Mordechai Himelfarb, Ariel University, Ariel, Israel; ^2^Department of Communication Disorders, Auditory Perception Lab in the Name of Laurent Levy, Ariel University, Ariel, Israel; ^3^Department of Gerontology, University of Haifa, Haifa, Israel; ^4^Baruch Ivcher School of Psychology, Reichman University, Herzliya, Israel; ^5^Department of Health Systems Management, Ariel University, Ariel, Israel; ^6^Meuhedet Health Services, Tel Aviv, Israel

**Keywords:** listening effort, pupillometry, speech recognition in noise, pupil dilation, school aged children

## Abstract

**Introduction:**

Children experience unique challenges when listening to speech in noisy environments. The present study used pupillometry, an established method for quantifying listening and cognitive effort, to detect temporal changes in pupil dilation during a speech-recognition-in-noise task among school-aged children and young adults.

**Methods:**

Thirty school-aged children and 31 young adults listened to sentences amidst four-talker babble noise in two signal-to-noise ratios (SNR) conditions: high accuracy condition (+10 dB and  + 6 dB, for children and adults, respectively) and low accuracy condition (+5 dB and + 2 dB, for children and adults, respectively). They were asked to repeat the sentences while pupil size was measured continuously during the task.

**Results:**

During the auditory processing phase, both groups displayed pupil dilation; however, adults exhibited greater dilation than children, particularly in the low accuracy condition. In the second phase (retention), only children demonstrated increased pupil dilation, whereas adults consistently exhibited a decrease in pupil size. Additionally, the children’s group showed increased pupil dilation during the response phase.

**Discussion:**

Although adults and school-aged children produce similar behavioural scores, group differences in dilation patterns point that their underlying auditory processing differs. A second peak of pupil dilation among the children suggests that their cognitive effort during speech recognition in noise lasts longer than in adults, continuing past the first auditory processing peak dilation. These findings support effortful listening among children and highlight the need to identify and alleviate listening difficulties in school-aged children, to provide proper intervention strategies.

## Introduction

Background noise poses a challenge for speech perception since it requires not only identification of the target speech, but also filtering from the noise; it is a critical everyday task that should be measured if a listener’s daily functioning is in question. Widely used clinical measures of peripheral hearing ability, such as the pure-tone audiogram and speech tests in quiet, cannot fully detect speech-in-noise difficulties (Holmes and Griffiths, 2019). The ability to recognize speech in noise relies upon a listener’s successful pairing of the acoustic–phonetic details from the bottom-up input with top-down linguistic processing of the incoming speech stream (Mattys et al., 2012; Moberly and Reed, 2019).

Speech perception can be assessed with a variety of tools and stimuli. Speech perception tests are typically applied to measure either intelligibility-the proportion of correctly repeated speech items, usually single words, or single sentences-or the intensity (in dB) required for a 50% accurate speech reception threshold (SRT). Speech perception tests based on word recognition are sensitive to the listener’s hearing ability (Mackersie, 2002), but have low real-world application, as we speak in more than just syllables or single words. In order to simulate everyday life, it is essential to use sentences that include a changing acoustic pattern over time (Bell and Wilson, 2001).

Studies show that school-aged children spend most of their time in school, listening in the presence of background noise. Previous studies have suggested that speech signal degradation due to unfavorable transmission conditions, such as background noise, has a more negative effect on children than on adults (Thorpe et al., 1989; Fallon et al., 2000; Wightman and Kistler, 2005; Valente et al., 2012). Children have more difficulties than do adults in understanding speech in noisy and reverberant environments, thus requiring more favorable signal-to-noise ratios (SNRs) to achieve adult-like performance (Nittrouer et al., 1990; Crandell and Smaldino, 2000; Crukley and Scollie, 2012). This may be due to the progressive development of children’s auditory system (Hall and Grose, 1990; Moore et al., 2001). Thus, children’s speech recognition abilities depend on their ability to separate speech from noise, and to benefit from fluctuations in background noise and binaural cues.

Children’s poor selective attention has high negative impact on auditory perception. This is reflected in higher susceptibility to informational masking in auditory signal detection tasks and more intrusions from distractor messages in dichotic listening tasks (Wightman and Kistler, 2005). However, studies show that the ability to recognize speech accompanied by noise is also related to cognitive functioning (such as working memory), executive functioning (Sullivan et al., 2015; von Lochow et al., 2018), and language development (Myhrum et al., 2016). These findings suggest that cognitive resources are involved when listening in noise. Indeed, studies have shown that sentence repetition robustly taps into long-term linguistic knowledge, such as vocabulary, grammar (Klem et al., 2015; Polišenská et al., 2015), and working memory (WM; Riches, 2012; Archibald et al., 2013). Linguistic knowledge and WM are still developing in school-aged children, with significant improvement occurring during these years (Camos and Barrouillet, 2011; Magimairaj and Montgomery, 2012; Camos and Barrouillet, 2014). However, while adults can employ cognitive resources for speech recognition when auditory stimuli are degraded (Sullivan et al., 2015), such resources are not yet fully matured among children. This suggests that, when needed, children and adolescents may be less able to employ cognitive resources than adults, making it more difficult to separate target speech from background noise (Osman and Sullivan, 2014; Sullivan et al., 2015). This, in turn, might lead not only to a decline in speech recognition in noise, as compared to adults, but also to a larger effort invested by children, when required to perform such a task.

Measuring the effort required for speech recognition has been the subject of numerous studies over the last decade. The topic has been investigated extensively in adults (Pichora-Fuller et al., 2016), but there are fewer studies in children (Rudner et al., 2018). This is despite the fact that children, like adults, experience challenging listening situations; the school classroom is a good example of this, given the likelihood of needing to perceive speech in the presence of noise (Sahlén et al., 2018). Effort while performing speech recognition in noise has mainly been studied among children using the dual task paradigm (DTP). The DTP is based on the concept that, when several processes compete for the same resources, performance will deteriorate (Norman and Bobrow, 1975; Navon and Gopher, 1979). The classic dual-task paradigm requires a participant to perform two tasks concurrently. One task is the primary task (auditory), and the secondary task is used as a competing task. Typically, listening effort (LE) is calculated as the difference in performance on the secondary task between the baseline condition and the dual-task condition (the concurrent performance of the tasks). However, this method has provided inconsistent results in children regarding performance accuracy and response time (RT) on the secondary task (Choi et al., 2008; McGarrigle et al., 2017a; Oosthuizen et al., 2020). These inconsistent findings may be explained either by the secondary task RTs being less stable in young children (under 12 years old) than in older children (Picou et al., 2017), or perhaps by school-aged children’s still developing abilities, thus contributing to a general variance in dual task performance (McGarrigle et al., 2019).

To measure the effort while performing speech recognition in noise, the current study utilizes pupillometry - a psychophysiological method of measuring pupil size that is considered an objective non-invasive measure of cognitive effort (Zekveld et al., 2010; Koelewijn et al., 2012; Zekveld et al., 2018). Pupil size has been shown to be sensitive to changes in cognitive resource allocation in a wide variety of tasks presented in different modalities (Zekveld et al., 2018) and pupillometry has become a frequently used measure of cognitive effort. Pupillometry is considered an index of effort in cognitive control tasks in general (van der Wel and van Steenbergen, 2018), and over the last decade, in LE in particular (Zekveld et al., 2010). In general, pupil size increases with increasing task demands as long as the individual stays engaged in the task (Granholm et al., 1996; Wendt et al., 2018). When task demands are beyond the participant’s ability, the pupil size will decrease, indicating a task that is too difficult for the participant to maintain engagement (Koelewijn et al., 2014; McGarrigle et al., 2017b; Wendt et al., 2018). The use of an eye tracker provides a high temporal (and spatial) resolution measure of pupil size changes thought to result from task difficulty (Kahneman and Beatty, 1966). Pupils dilate in response to increases in arousal and mental effort, either triggered by an external stimulus or spontaneously. Pupillometry is now attracting considerable interest in the auditory modality as pupil diameter size can provide an objective index of the sensory and cognitive challenge of processing a target speech stream in the presence of distracting speakers (Zekveld et al., 2010; Wang et al., 2018).

One crucial advantage of pupillometry over behavioral or subjective measures of listening effort is that pupil size varies during the task, continuously tracking changes in cognitive resource allocation over time. Timing is an essential part of understanding listening effort because speech demands rapid auditory encoding as well as cognitive processing distributed over time, rather than being deployed all at once at the end of a stimulus (Winn et al., 2018). Conversely, behavioral measures tend to reflect changes that occur after the speech processing phase (Peelle, 2018). During the process of effortful listening pupillary response might measure a cognitive dimension different from the one measured by reaction times and self-ratings. Different potential measures of effort tap into different underlying cognitive dimensions (Alhanbali et al., 2019). In the current study, we tested pupillometry during a speech in noise task while examining accuracy scores in each task.

Pupillometry is suitable for assessing the performance of children, and studies have shown that task-evoked pupil response appears to be sensitive to changes in LE in children (Steel et al., 2015; Brännström et al., 2021). Nevertheless, only few studies to date used pupillometry to examine mental effort in children while performing auditory tasks (Steel et al., 2015; Lum et al., 2017; McGarrigle et al., 2017a). These studies showed increase in pupil dilation when listening conditions were challenging.

Studies measuring pupil size among children need to overcome logistical constraints such as stabilized head position, lack of sustained attention, and lack of patience for a very plain, unstimulating visual field that would certainly make pupil measurements in young children difficult (Winn et al., 2018). Another difficulty is to compare children’s pupil sizes to those of adults, due to changes in absolute pupil diameter that occur over a lifetime. Children show larger absolute pupil sizes than do adults: During the first 10 years of age, absolute pupil size increases; at ages 11–15 it plateaus; and afterwards it slowly but consistently shrinks with increasing age (MacLachlan and Howland, 2002; Eckstein et al., 2017).

Pupil size in speech perception tasks is measured from the time of stimuli presentation, throughout the listening phase, and until after a response is received. However, most previous studies on children explored specific time points during stimuli presentation and the listening phase (Steel et al., 2015; McGarrigle et al., 2017a).

Therefore, there is no information on continuous processes underlying listening and speech recognition. To fill this gap in knowledge, in the present study, we analyzed the continuous process of speech recognition from listening, through retrieval, to responding, in order to examine the assumption that listening in noise requires a higher cognitive effort in children than in adults.

To achieve this goal, we used a classic speech perception paradigm of sentences accompanied by four-talker babble noise (4TBN). Two different mid-level difficulty conditions were used, each having been adapted to achieve similar identification rates by normal hearing children and adults. Participants listened to the sentences and then repeated them, while pupil size was collected continuously (every 25 ms) from stimulus delivery until after the response.

## Materials and methods

### Participants

Thirty children (17 girls; age range = 7.5–10.5 years; mean age = 8.5 years) and thirty-one young adult undergraduate university students (15 females; age range = 20.5–34; mean age = 27.9 years) were recruited for the current study. This sample size corresponds to previous studies that measured pupillometry of children or adults (Steel et al., 2015; Russo et al., 2020; Seifi Ala et al., 2020; Winn and Teece, 2021). All the participants, native Hebrew speakers, were screened for bilateral normal hearing, and had: (1) hearing thresholds of pure tones under 20 dB in 0.5, 1, 2, and 4 kHz; (2) normal or corrected-to-normal vision; and (3) no reported history of attention deficit disorder or learning disabilities based on parental report (for children) or self-report (for adults). For the children, all participants had normal speech, language, and motor development, and no reported history of ear infections as confirmed by parental report.

The study was approved by the institutional ethics committee, and all adult participants and the children’s parents signed an informed consent to take part in the experiments. For compensation, they received the equivalent of $25 for participation.

### Stimuli

Fifty-six sentences (twenty-eight in each SNR condition) were taken from a pool of Hebrew sentences created according to the Hearing In Noise Test (HINT) principles (Nilsson et al., 1994). The sentences used in the study were carefully selected to be suitable for children aged 7–11, considering their linguistic knowledge and abilities. These sentences, which are 5–7 syllables in length and rated at the first-grade reading level, were specifically chosen to align with the cognitive capabilities of the child participants. Sentences in each list were similar in length and presented at 65 dBSPL by a recorded female voice in the presence of competing 4TBN. The 4TBN includes two male and two female talkers. The recordings were normalized to have the same root mean square (RMS) amplitudes and were combined into a single recording of four-talker babble noise. The 4TBN was presented in two different signal-to-noise-ratios (SNRs). Sentences were presented in blocks of continuous noise, one block for each SNR. Each block included twenty-eight sentences, resulting in two lists (fifty-six sentences) for each participant. Blocks were randomized between subjects using a Matlab script. Since children require more favorable SNRs than adults, we performed a pilot test in which we tested an additional ten children (mean age = 9 years) and fifteen young adults (mean age = 28 years) and found two levels of SNRs that provided a similar identification percentage among each group.

### Apparatus

Sentences accompanied by different SNRs for each group were produced using a Matlab script. The speech was presented at a fixed level of 65 dBSPL, and the noise level varied to create the relevant SNR values. The sentences were presented in Tobii Pro Lab version 1.95 eye-tracking software, with auditory delivery via an RCF AYRA 5 Active 5″ 2-Way Professional Studio Monitor Speaker located 60 to 65 cm from the front of participants’ heads. Pupil size was measured using a video-based desktop-mounted Tobii Pro X3 − 120 eye tracker with a pupil size sampling rate of 40 Hz.

### Procedure

Upon arrival, adult participants and the children’s parents signed an informed consent, and the children gave oral consent to participation. Following that, participants performed a hearing screening test using an AD229B audiometer (Interacoustics) with supra-aural headphones. The experiment was conducted in a single session that lasted approximately 25 min in a room lit to 300 lux as verified by a lux meter (Testo 540 lux meter). Participants were positioned in a chair at a distance of 60–65 cm from the center of a computer screen (their nose was parallel to the central eye tracking sensor) and instructed to sit in a position they could comfortably maintain for at least 25 min. All participants received verbal encouragement during the test.

**Figure 1:**
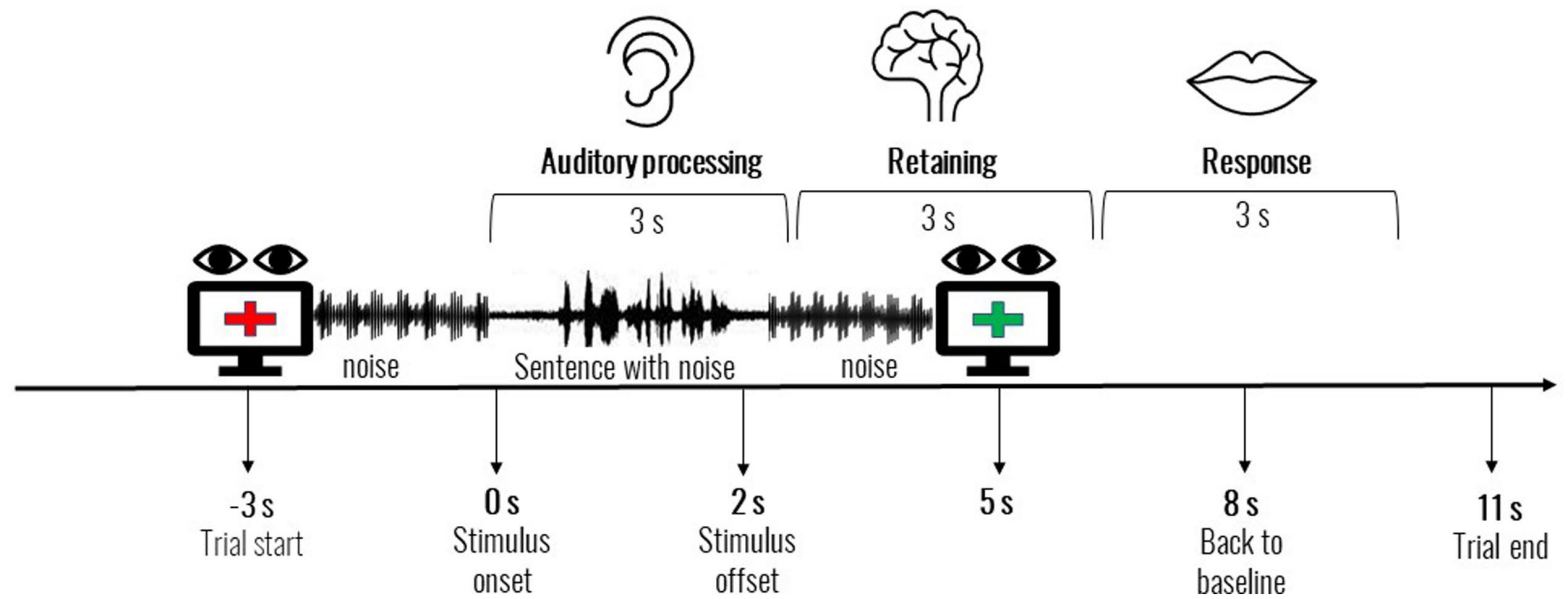
Experimental design and trial structure. The listener views a fixated on a red cross and three seconds after the offset of the sentence, the cross on the screen turns green to elicit the verbal response.

Prior to testing, participants underwent a short calibration procedure (with eight points of calibration). During the test, a red fixation cross was presented at the center of a computer screen on a grey colored background (defined as [102102102] in RGB color space). Participants were instructed to verbally repeat the sentences they heard when the fixation cross turned from red to green. During testing, each sentence was preceded and followed by 3 s of four-talker babble noise to permit baseline pupil diameter measurement. After each sentence was aurally presented, the red fixation cross changed to green, in equal luminance, signaling that the response should be given. All participants reliably detected the color change. The green fixation cross was presented for 6 s: 3 s to allow participants to respond, and 3 s for pupil diameters to return to baseline (See Figure 1). Responses were transcribed and coded by a trained experimenter. Scoring was calculated as the percentage of correct words repeated by the subject out of the total number of words in each list of sentences.

### Pupil data pre-processing

Pupil size data was extracted from the Tobii eye tracker (in mm) and processed using CHAP software (Hershman et al., 2019). Outlier samples with *z*-scores larger than 2.5 (based on means and standard deviations calculated for each trial) were removed. Following previous studies (Hershman et al., 2019; Hershman and Henik, 2019), all trials with more than 30% missing values were excluded from the analysis for each participant, and participants with fewer than 20 valid trials in each condition were excluded. Thus, 12 participants of the initial sample were excluded from the analysis. Eye blinks were detected using Hershman, Henik, and Cohen’s algorithm (Hershman et al., 2018), and missing values were completed using linear interpolation (Hershman and Henik, 2019).

### Adjustment for differences in pupil size

As previously mentioned, children have larger absolute pupil size relative to adults (MacLachlan and Howland, 2002; Eckstein et al., 2017). In the present study, the pupil dynamic range for the children was 3.26–5.92 and for the adults was 2.63–4.38 mm. To avoid age differences as a result of differences in the pupil dynamic range, we normalized the participants’ pupil size according to their pupil dynamic range during the task: 100 × (d_M_ − d_min_)/(d_max_ − d_min_), whereas d_M_ is the participant’s pupil size at each time point, and d_min_ and d_max_ are the minimum and maximum pupil sizes during the task (Ayasse et al., 2017). Therefore, the analyses were conducted on normalized pupil diameter data.

### Statistical analysis

Behavioral speech perception accuracy was analyzed using a mixed-model repeated-measures ANOVA, with Accuracy Level SNR (high, low) as a within-subjects variable, and Group (children, adults) as a between-subjects variable. Analyses of pupillometry data were conducted separately for the different timeframes: auditory processing (starting at sentence onset and ending 3,000 ms post sentence onset), retaining (3,000–6,000 ms) and response (6,000–9,000 ms). All analyses used MLM, mixed general linear modeling (SPSS Statistics 26, IBM Corp.), with age-group (X2: children, adults) as between-participants variable and SNR (X2: high accuracy, low accuracy) as within-participant variable. The dependent variable was normalized change in pupil diameter. The time course of pupil dilation was analyzed using Growth Curve Analysis (GCA; Mirman et al., 2008). We used second (cubic) or third (quadratic) orthogonal polynomial, depending on the best-fit models (as gauged by BIC). In our models age-group and SNR were treated as fixed effects and intercept as random effect, as multiple random effects in higher terms prevented the models from converging. The reference (default) was the adults group and the high accuracy. The models used the following equation:


VALUEij=π0j+π1j∗(TIMEij)+π2j∗(TIME^2ij)+π3j∗(TIME^3ij)+rij

$$\begin{array}{c} \pi 0j=\beta 00+\beta 01^{*}\left(\mathrm{SNRj}\right)+\beta 02^{*}\left(\mathrm{GROUPj}\right)\\ +\beta 03^{*}\left({\mathrm{SNR}}^{*}\mathrm{GROUP}\right)+u0j \end{array}$$

$$\pi 1j=\beta 10+\beta 11^{*}\left(\mathrm{SNRj}\right)+\beta 12^{*}\left(\mathrm{GROUPj}\right)+\beta 13^{*}\left({\mathrm{SNR}}^{*}\mathrm{GROUP}\right)$$

$$\pi 2j=\beta 20+\beta 21^{*}\left(\mathrm{SNRj}\right)+\beta 22^{*}\left(\mathrm{GROUPj}\right)+\beta 23^{*}\left({\mathrm{SNR}}^{*}\mathrm{GROUP}\right)$$

$$\pi 3j=\beta 30+\beta 31^{*}\left(\mathrm{SNRj}\right)+\beta 32^{*}\left(\mathrm{GROUPj}\right)+\beta 33^{*}\left({\mathrm{SNR}}^{*}\mathrm{GROUP}\right).$$

## Results

### Behavioral analysis

**Figure 2:**
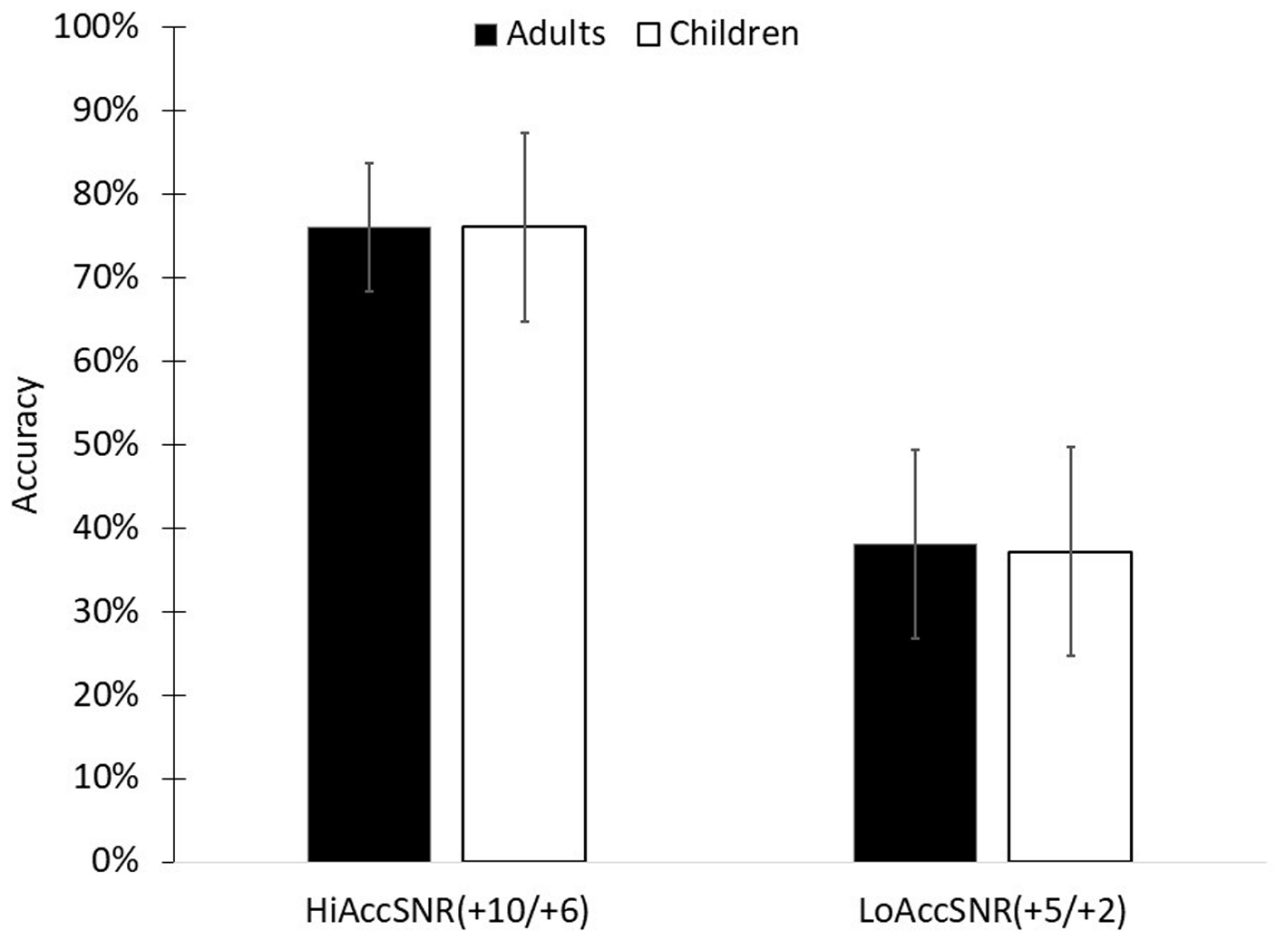
Accuracy rates among children and adults for high and low accuracy SNRs HiAccSNR = High Accuracy Signal to Noise Ratio (+10 dB/+6 dB); LoAccSNR = Low Accuracy Signal to Noise Ratio (+5 dB/+2 dB).

The SNRs values,that were found in a pilot test, for the children were: +10 dB and + 5 dB, and for adults were: +6 dB and + 2 dB. The high accuracy SNRs (+10 dB and + 6 dB) resulted in accuracy rates of 76.06% (SD = 11.31%) for the children and 76.00% (SD = 7.69%) for the adults; the low accuracy SNRs (+5 dB and + 2 dB) resulted in accuracy rates of 37.19% (SD = 12.47%) for the children and 38.08% (SD = 11.23%) for the adults (Figure 2). There was a main effect for Accuracy Level SNR (*F*(1,39) = 508.466, *p* < 0.001, partial *η*^2^ = 0.929), but no effect for Group (*F*(1,39) = 0.008, *p* = 0.931, partial *η*^2^ = 0.000) and no Accuracy Level SNR X Group interaction (*F*(1,39) = 0.006, *p* = 0.936, partial *η*^2^ = 0.000).

### Pupillometry

**Figure 3:**
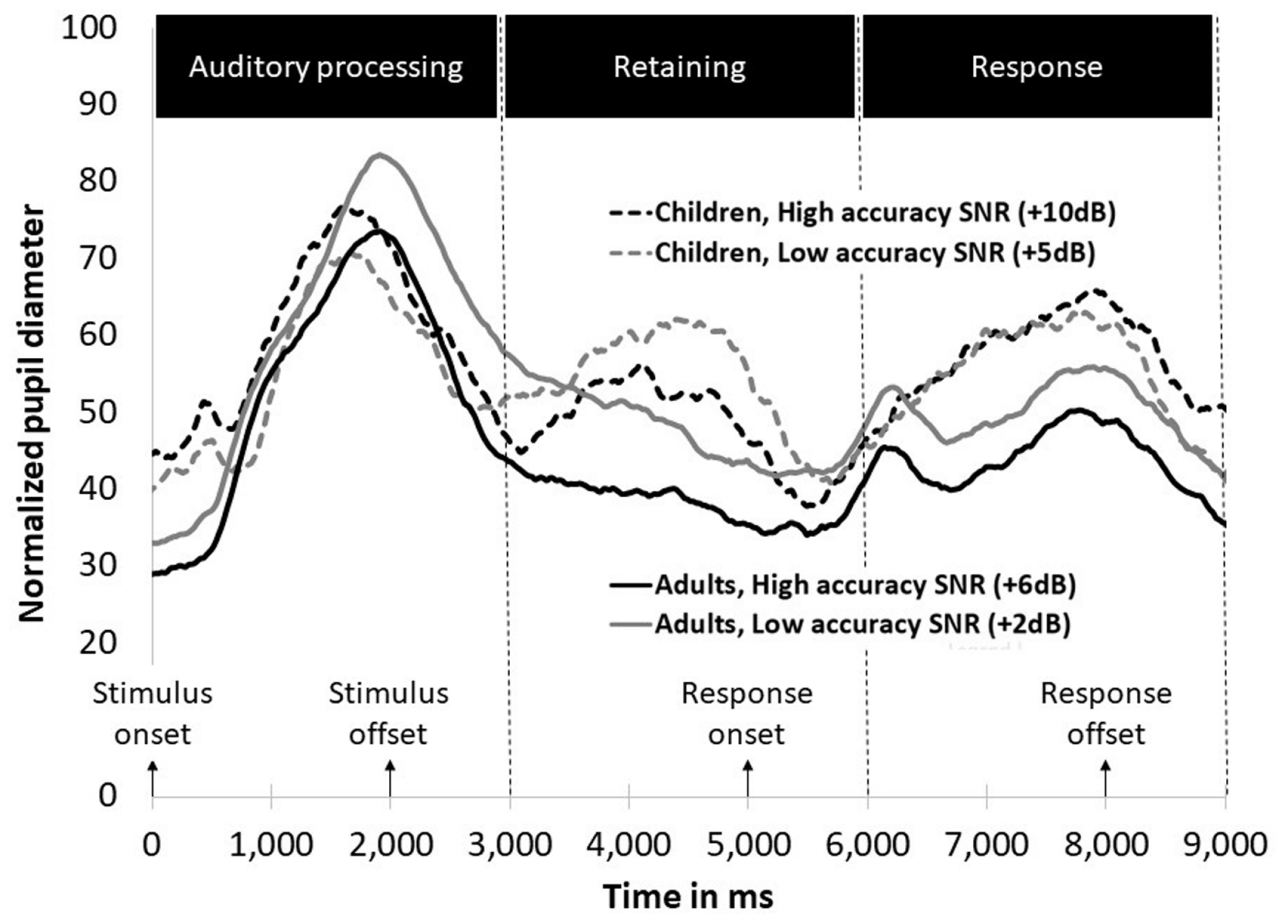
Mean normalized pupil diameter for two accuracy level SNRs during the speech perception test timing for children and adults.

Figure 3 illustrates changes in pupil dilation over time split by group (children and adults) and SNRs (high and low accuracy).

*Timeframe 1*: Table 1 displays the output of the MLM analysis conducted for the auditory processing phase (phase 1). A significant main effect for Group was found for the growth terms (cubic and quadratic), signifying more pupil dilation for the adults. The graph demonstrates that this difference stems mostly from adults’ pupil dilation in the low-accuracy condition. No significant main effect for SNR was found. A significant SNR X Group interaction was found for the intercept only. A follow up analysis conducted separately for children and adults showed that SNR affected performance at the intercept only for the adults (*t* = −3.313, *p* < 0.001) and not for the children (*t* = 1.344, *p* = 0.179).

**Table 1:** Results of a GCA conducted for the auditory processing timeframe.

Auditory processing		Growth curve analysis			
Effects		Estimate	SE	*t*	*p*
SNR	Intercept	2.965	1.978	1.498	0.134
	Linear	0.004	0.005	0.851	0.395
	Cubic	−1.158E-6	4.453E-6	−0.260	0.795
	Quadratic	0.000	0.000	−0.316	0.752
Group	Intercept	−8.122	5.540	−1.466	0.150
	Linear	0.001	0.008	0.205	0.828
	Cubic	1.146E-5	4.435E-6	2.584	0.010**
	Quadratic	−3.403E-9	0.000	−3.853	0.001**
SNR*Group	Intercept	−8.593	2.534	−3.391	0.001**
	Linear	0.002	0.007	0.392	0.695
	Cubic	−6.066E-6	5.703E-6	−1.064	0.287
	Quadratic	1.501E-9	1.249E-9	1.202	0.229

* *p* < 0.05.

** *p* < 0.01.

*Timeframe 2*: Table 2 shows the results of the MLM analysis conducted for the retaining phase (phase 2). Significant main effects were found for Group for the intercept and the growth terms, suggesting increased pupil dilation for children, whereas for the adults, pupil size was decreased. A significant main effect of SNR was found for the growth terms, showing that in the low accuracy condition, the size of the pupil was larger than in the high accuracy condition. A significant Group X SNR interaction was found for both the intercept and the growth terms. Visual inspection of the graph shows that adults’ pupil size decreased while the children’s pupil size increased. Follow up interaction conducted separately for each group revealed SNR effects for adults in the intercept and the growth terms, indicating larger pupil size for the low-accuracy condition (*t* = −12.634, *p* < 0.001, *t* = 3.003, *p* = 0.002 for the intercept and the linear terms, respectively). For children, SNR affected only the growth terms, as can be visually seen in a large increase in pupil dilation for the low accuracy condition (*t* = −1.651, *p* = 0.099, *t* = −3.509, *p* < 0.001 for the intercept and linear terms, respectively).

**Table 2:** Results of a GCA conducted for the retaining timeframe.

Retaining		Growth curve analysis			
Effects		Estimate	SE	*t*	*p*
SNR	Intercept	−2.386	1.363	−1.750	0.080
	Linear	0.007	0.002	−3.718	0.001**
	Cubic	2.816E-6	6.775E-7	4.156	0.001**
Group	Intercept	9.041	3.906	2.315	0.025*
	Linear	−0.028	0.004	−6.142	0.001**
	Cubic	9.269E-6	1.519E-6	6.100	0.001**
SNR*Group	Intercept	−12.154	1.746	−6.960	0.001**
	Linear	0.013	0.002	4.882	0.001**
	Cubic	−3.751E-6	8.677E-7	−4.323	0.001**

* *p* < 0.05.

** *p* < 0.01.

**Figure 4:**
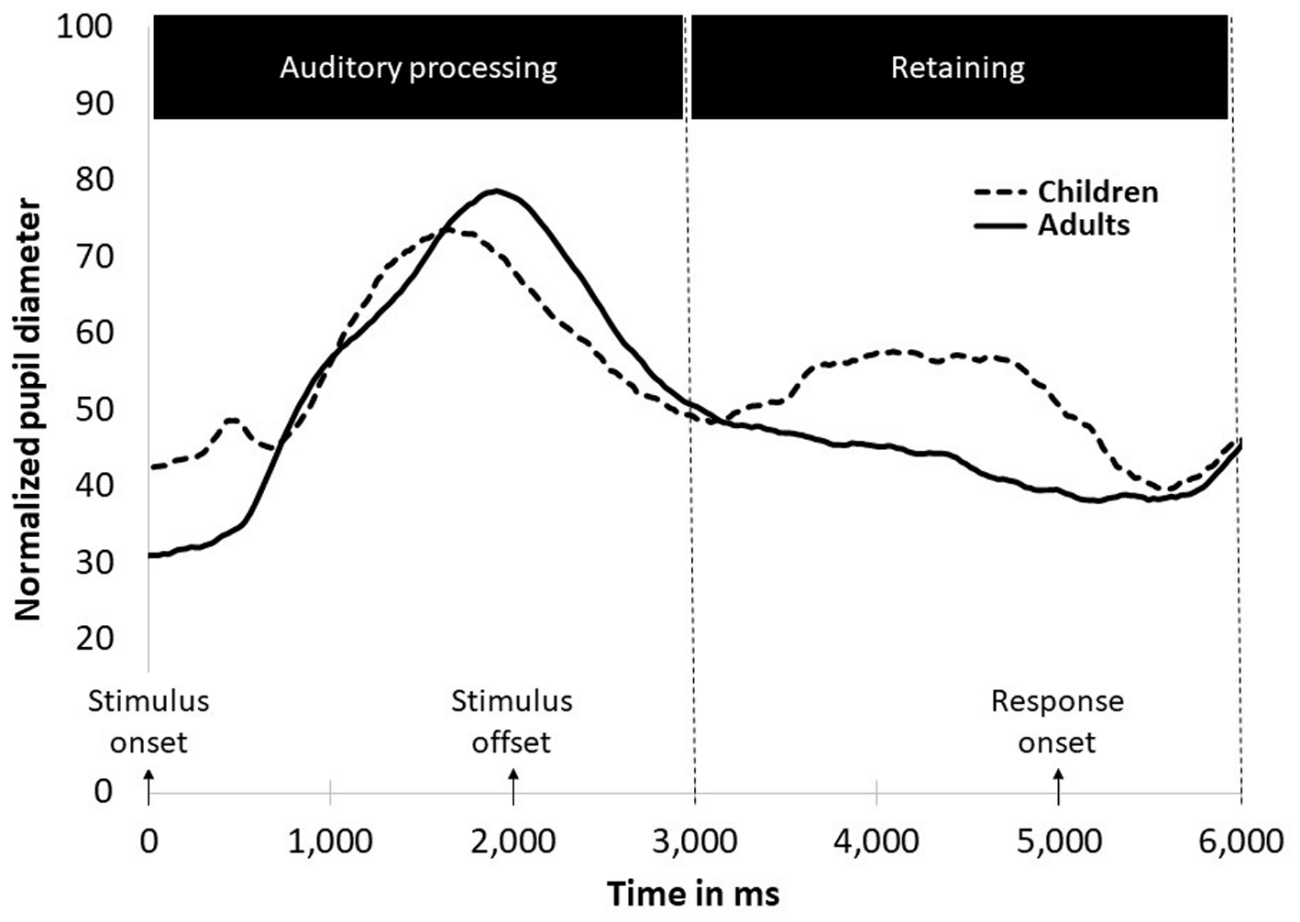
Pupil dilation in the auditory processing and retention timeframes averaged across SNR conditions.

Figure 4 presents pupil dilation in the auditory processing and retention timeframes averaged across SNR conditions for children and adults. Investigation of Figure 4 indicates that in timeframes 1 and 2 the adult group exhibited one pupil dilation peak in the auditory processing timeframe and the children group exhibited two dilation peaks - in the auditory processing and in the retention timeframes. To validate this observation, we performed an analysis of the mean pupil dilation for each group across both timeframes (1,000–7,000 ms) and found that overall, the children group exhibited more pupil dilation than the adult group, *F* (1, 70,183) = 3.927, *p* = 0.051.

*Timeframe 3*: Results of the MLM analysis conducted for the response phase are presented in Table 3. When participants were responding to the stimuli, no significant effect for SNR was found. A significant main effect was found for Group on the growth terms, showing larger pupil dilation for the children’s group. A significant Group X SNR interaction was found for the intercept. Follow up analyses conducted separately for the children and the adult groups found that only the adults were affected by SNR (*t* = −5.434, *p* < 0.001 and *t* = 0.938, *p* = 0.348 for the intercept for the adults and children, respectively).

**Table 3:** Results of a GCA conducted for the response timeframe.

Response		Growth curve analysis			
Effects		Estimate	SE	*t*	*p*
SNR	Intercept	1.812	1.708	1.061	0.289
	Linear	−0.004	0.004	−0.927	0.354
	Cubic	3.281E-6	3.845E-6	0.853	0.394
	Quadratic	0.000	0.000	−0.425	0.671
Group	Intercept	9.061	4.760	1.903	0.063
	Linear	−0.038	0.007	−5.018	0.001**
	Cubic	2.274E-5	3.983E-6	5.711	0.001**
	Quadratic	−3.685E-9	0.000	−4.831	0.001**
SNR*Group	Intercept	−10.008	2.188	−4.574	0.001**
	Linear	0.008	0.006	1.397	0.163
	Cubic	−5.670e-6	4.924e-6	−1.151	0.250
	Quadratic	0.000	1.078e-9	0.707	0.479

* *p* < 0.05.

** *p* < 0.01.

## Discussion

This study conducted temporal analysis of pupil dilation among children and young adults during a speech recognition task accompanied by 4TBN, to measure possible differences of effort underlying performance. Two key findings emerged from the study: (1) Behavioral speech recognition results showed significant differences between high and low SNRs among both children and adults and (2) Significant differences between children and adults were found in the three phases of pupil dilation across the task timeline (auditory processing, retaining, and response), as detailed below.

When comparing cognitive effort between children and adults during speech perception, a possible confounding factor may be differences in the actual difficulty of the task. Therefore, we provided each group with different SNRs, because children require more favorable SNRs than do adults in order to achieve the same accuracy (Nittrouer et al., 1990; Crandell and Smaldino, 2000; Crukley and Scollie, 2012). Indeed, these different SNRs produced similar intelligibility scores between the groups.

The task of speech recognition in noise required participants to listen to each sentence presented, and then to respond by repeating it. Accordingly, pupil dilation is observed, for both children and adults, when stimulus was presented and processed. During the task, both groups exhibited pupil dilation in the auditory processing phase, however adults exhibited larger pupil dilation than children. In the adults’ group, the pupil dilation was larger when the recognition score was low (approximately 40%) and lower when the score was high (approximately 80%).

This finding is in line with previous studies that demonstrated maximum pupil dilation with SNRs producing approximately 50% correct sentence recognition and relatively smaller pupil dilation at very high SNRs (producing accuracy scores more than 70%) (Ohlenforst et al., 2018; Wendt et al., 2018). This is in line with models of LE such as the Framework for Understanding Effortful Listening (FUEL). According to this model, speech recognition in noise is modulated by task demands (external effect) and motivation (internal effect). When the task demands are high but not too much, and the motivation is high, one is typically more engaged (Pichora-Fuller et al., 2016).

In the children group, however, there was no different pupil dilation in the presence of different intelligibility scores, within the auditory processing phase. This suggests children did not exert more mental resources in the low SNR. It accords with studies that explored listening effort in children with a dual task paradigm. They also showed that adding or changing the level of background noise did not affect their effort (Hicks and Tharpe, 2002; Howard et al., 2010; Picou et al., 2017).

The second phase of dilation, which appeared only in the children’s group and larger in the low SNR, could still be related to the children’s initial processing of the stimulus. Challenging tasks sometimes demand not only higher peak effort but also continuous and prolonged effort, suggesting that there is meaningful data to be explored in the pupillary response that appears after a sentence is heard (Winn and Moore, 2018). Indeed, when we analyzed the two phases together, children showed significantly overall larger and prolonged dilation compared to adults, which was somewhat obscured while analyzing only the first phase.

Notwithstanding, the second phase may reflect a separate process of retaining the stimulus. This second phase of pupil dilation corresponds in timing to working memory maintenance, theorized to be exerted during a two-second silent period after listening to a stimulus (Sternberg, 1966; Baddeley, 2000). Indeed, previous pupillometry studies found continued linguistic processing that was reflected in dilation during the retention window and showed that the retention interval is a good reflection of the cumulative memory load (Piquado et al., 2010; Winn and Litovsky, 2015).

The working memory capacity of school aged children is less developed in comparison to that of adults (Kambara et al., 2018). The literature shows that WM is still developing in school-aged children, and significantly improves during these years (Camos and Barrouillet, 2011; Magimairaj and Montgomery, 2012; Camos and Barrouillet, 2014). Indeed, despite comparable intelligibility scores, pupillometry data was different, with a “second phase” of dilation for children only. This second phase may be the result of the children’s still-underdeveloped WM that possibly required mental effort for children during the retention phase, but not for adults. This idea is supported by the Ease of Language Understanding (ELU) model. It proposes that distorted information, such as when speech is accompanied with noise, mismatches with representation stored in long-term memory (LTM) representation. This mismatch causes processing to be slow and effortful, recruiting more cognitive resources, such as WM and attention for understanding speech. Moreover, it suggests that WM is one of the main cognitive functions that becomes involved when demands on explicit processing are involved (Rönnberg et al., 2018). According to the ELU theory, individuals with high WM capacity, as typically observed in adults, are generally more efficient at allocating cognitive resources for processing speech in noise compared to those with lower WM capacity, such as children (Rönnberg et al., 2008, 2013). The results of the current study support this claim and demonstrate cognitive effort among the children when processing speech in noise (retention phase), while such effort is not demonstrated by the adults.

Adults did not show a separate second dilation phase as shown by the children. In the second phase, our data showed that adults’ pupil size gradually decreased. Previously, hearing impaired participants showed continuous high dilation in this phase (Winn, 2016; Winn and Moore, 2018). This was interpreted as reflecting prolonged auditory perception and slower release of effort. This can also explain the current data, but with a lesser effort, since the participants had normal hearing. The prolonged dilation might also reflect an effort during working memory maintenance, as we suggested had happened for children, but again, with lesser effort, due to their mature WM capacity.

In the third phase, pupil dilation of children was larger compared to adults with no effect of the SNR. To our knowledge, this phase has not been studied previously among children; however, the results here are not surprising. Since participants are required to respond verbally to repeat the sentences heard, it seems logical that this cognitive and motoric action would be reflected in mental effort. Thus, pupil dilation in this phase seems to represent the preparation for the motor response and the actual motor response. The analysis comparing pupil size between children and adults showed age differences in pupil dilation in this phase, with larger dilation among the children. This comparison should be regarded cautiously, as a general difference in the pupil dilation range can still be observed between children and adults, despite normalization. However, the differences in pupil dilation in this phase may also be explained by age differences in cognitive effort. Several studies have showed that although large gains in speech, motor skills, and articulatory timing control are made during the first few years of life, adult-like control is not achieved until mid-adolescence (Kent and Forner, 1980; Smith and Goffman, 1998; Green et al., 2000; Smith and Zelaznik, 2004). As such, children’s preparation for motor responses might recruit more effort than adults’.

The present study is one of the few testing LE among children. To best address this aim, we used pupillometry as a means to explore the mental effort that underlies performance. Other studies have used RT to measure mental effort, under the assumption that a slower RT reflects higher mental effort (Choi et al., 2008; McGarrigle et al., 2017a; Oosthuizen et al., 2020), while others have utilized decreased performance on a secondary task of dual task paradigms to reflect mental effort (Picou et al., 2017). While both are reasonable, valid measures of mental effort, the advantage of pupillometry is that it can provide information about cognitive effort on a moment-to-moment basis, and not only the sum or average of the overall outcome of cognitive processing, as done by other methods.

While most previous pupillometry studies focus on cognitive effort during the first listening phase following presentation of speech stimuli (McGarrigle et al., 2017a,b, 2021), we were able to track effort throughout the processing phase, and until after the response. Indeed, the moment-to-moment information on cognitive effort provided by this methodology resulted in the main findings of the current study. It led to the identification of differences between children and adults in the effort put into processing speech. This finding is especially important considering the behavioral findings that have shown similar intelligibility and, therefore, highlights the importance of considering mental effort in addition to behavioral performance. This was previously observed by others who showed differences in mental effort despite similar behavioral performance (McGarrigle et al., 2017a).

Therefore, we recommend future research to look at moment-to-moment information about cognitive effort in order to maximize the understanding of mental effort during speech perception in all kinds of populations, such as hearing-impaired or aging adults. In addition, as our study utilized only an identification task, future studies can also examine higher levels of processing by utilizing an auditory comprehension task.

In sum, the fact that school-aged children invest more effort during retention and response phases of speech perception should evoke consideration of listening effort as the first marker of fatigue. Knowledge of physiological mechanisms that support effortful listening could help to identify and eventually alleviate listening difficulties in school-aged children. A noisy environment is challenging and may have a negative effect on learning and academic achievement of children. Efforts to reduce noise in educational settings for children could greatly enhance student learning and reduce fatigue. As audiologists, it is common to come across patients that report difficulty and fatigue while listening, however the standard audiology test battery does not reflect these reported difficulties. As a result, the complaints are not captured in the current audiology testing. This points to listening effort-induced fatigue as an additional factor in children’s performance and should be considered in additional populations.

## Data availability statement

The raw data supporting the conclusions of this article will be made available by the authors, without undue reservation.

## Ethics statement

The study involving human participants were reviewed and approved by the institutional ethics committee of ariel university. Written informed consent to participate in this study was provided by the participants’ and legal guardian/next of kin.

## Author contributions

AT-M designed and performed the experiments, processed the data, and wrote the manuscript content. LF designed the experiments, analyzed the data, and wrote the manuscript content. TH-A analyzed the data and wrote the results. RN-G designed the experiments and provided the critical manuscript revisions. RT-S designed and performed the experiments, analyzed data, and wrote the manuscript content. All authors contributed to the article and approved the submitted version.

## Conflict of interest

The authors declare that the research was conducted in the absence of any commercial or financial relationships that could be construed as a potential conflict of interest.

## Publisher’s note

All claims expressed in this article are solely those of the authors and do not necessarily represent those of their affiliated organizations, or those of the publisher, the editors and the reviewers. Any product that may be evaluated in this article, or claim that may be made by its manufacturer, is not guaranteed or endorsed by the publisher.
